# Modeling Stem Cell Myogenic Differentiation

**DOI:** 10.1038/srep40639

**Published:** 2017-01-20

**Authors:** Rajiv S. Deshpande, Alexander A. Spector

**Affiliations:** 1 Department of Biomedical Engineering, Johns Hopkins University, Baltimore, MD 21205, USA

## Abstract

The process of stem cell myogenesis (transformation into skeletal muscle cells) includes several stages characterized by the expression of certain combinations of myogenic factors. The first part of this process is accompanied by cell division, while the second part is mainly associated with direct differentiation. The mechanical cues are known to enhance stem cell myogenesis, and the paper focuses on the stem cell differentiation under the condition of externally applied strain. The process of stem cell myogenic differentiation is interpreted as the interplay among transcription factors, targeted proteins and strain-generated signaling molecule, and it is described by a kinetic multi-stage model. The model parameters are optimally adjusted by using the available data from the experiment with adipose-derived stem cells subjected to the application of cyclic uniaxial strains of the magnitude of 10%. The modeling results predict the kinetics of the process of myogenic differentiation, including the number of cells in each stage of differentiation and the rates of differentiation from one stage to another for different strains from 4% to 16%. The developed model can help better understand the process of myogenic differentiation and the effects of mechanical cues on stem cell use in muscle therapies.

Effective models have recently been proposed for a variety of cells under different conditions where mechanical factors are involved. They include analyses of spreading on patterned substrates^1^, alignment under cyclic load^2^,^3^, mechanotransduction under applied shear forces^4^, deformation under 3-D flow forces^5^, force generation with 3-D tissue^6^, etc. However, the modeling of stem cell mechanobiology, where mechanotransduction converges with cell differentiation, remains less developed. For stem cell differentiation, the mechanical factors are of primary importance because they transform into cells where such factors are part of the cell microenvironment^7^,^8^,^9^,^10^. Moreover, it has been recognized that factors such as cell area^11^ substrate stiffness^12^, extracellular matrix (ECM) viscoelasticity^13^, and surface topography^14^,^15^ can be used as tools to direct and optimize stem cell differentiation. A number of stem cells, including satellite cells^16^, bone marrow stem cells^17^, and induced pluripotent stem cells^18^, have shown a potential for skeletal muscle treatment. One promising approach is related to adipose-derived stem cells (ASCs) because they are abundant and easily accessible in the body of a patient^19^. The mechanical factors can significantly affect ASC myogenesis^20^.

Huri *et al*. have recently shown that the application of strains to the myogenic environment significantly enhances the outcome of ASC differentiation^21^,^22^. To better understand this effect on stem cell myogenesis, we have proposed a phenomenological model^23^ where the strain effect was incorporated through the experimental data of Huri *et al*.^22^ for the static (no applied strains) and dynamic (strain magnitude of 10%) cases. However, the biological mechanisms of the strain effect and stem cell differentiation remained to be further developed.

In the present paper, we consider stem cell myogenesis and focus on its differentiation part (Fig. 1a) as well as on the mechanism of the strain effect. We add a transcription factor, myogenin, into consideration and model the late factors, MyoD, myogenin, and MHC, as a transcription network. We interpret the strain effect via a strain-generated signaling molecule that affects the transcriptional activity of the MyoD and myogenin factors (Fig. 1b). As a result, the transcription factors and the applied strain enter the model via saturating Michaelis-Menten functions instead of linear functions in our previous model^23^. Finally, we determined the optimal differentiation parameters of the model by fitting the available experimental data for ACSs subjected to the strain of 10% magnitude^22^ and predict the differentiation kinetics for different strains.

## Results

### Model of stem cell myogenic differentiation

**Figure 1:**
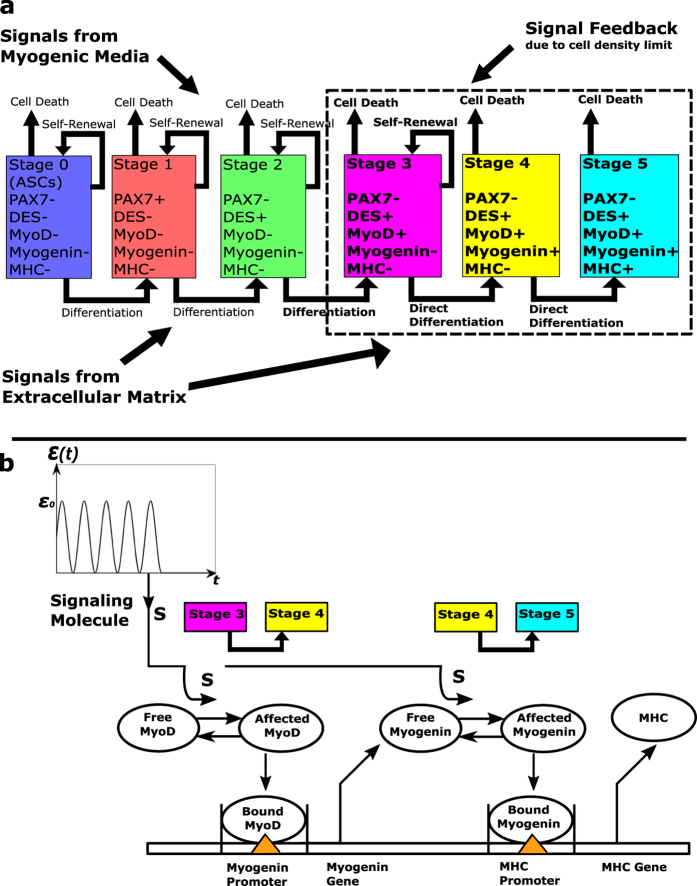
Conceptual model of stem cell myogenesis. (**a**) Six stages and the expression of myogenic factors. The first three stages occur via asymmetric cell division, and the latest three stages (shown within the dashed line) proceed through direct differentiation. The multi-stage process of stem cell myogenesis is affected by external signaling from the myogenic medium, extracellular matrix (strain effect), and cell-cell interaction if a cell density threshold is reached. (**b**) The proposed mechanism of stem cell myogenic differentiation associated with the interaction among the transcription factors, MyoD and myogenin, and the late myogenic factor, MHC. The strain effect is interpreted as strain-generated signaling molecule, S, that affects the transcriptional activity of MyoD and myogenin.

The focus of our model of stem cell myognenic differentiation is the kinetics of expression of myogenic factors, MyoD, myogenin and MHC, and the effect of the applied strain. We formulate our model in terms of the number of cells in the distinct stages of myogenesis which are determined by particular combinations of the expressed factors (Fig. 1a). In terms of the earlier (pre-differentiation) part of the myogenic process, we assume that it occurs via the mechanism of asymmetric division and results in the first transcription factor, MyoD reaching a threshold necessary for further differentiation^23^. This threshold is considered as independent of the applied strain but the moment of time when the threshold is reached is strain-dependent. The equations for the earlier stages’ kinetics (n_0_, n_1_, and n_2_) are not discussed in the present paper, although the values of n_o_, n_1_, and n_2_ affect the cell density feedback factor determined by the total number of cells. In addition, the cells in stage 2 contribute to the number of cells in stage 3 through asymmetric division. Wherever the characteristics of earlier stages 0, 1, and 2 enter the current model of stem cell myogenic differentiation or are graphically presented they are treated according to Deshpande *et al*.^23^.

The kinetics of stem cell myogenic differentiation can be described by the following equations


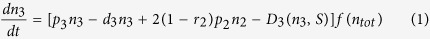



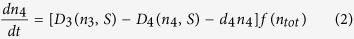



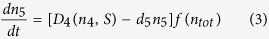


Here, the right-hand sides on equations (1, 2, 3) give the sums of fluxes that determine the rates of the cell numbers in stages 3, 4, and 5, respectively. The terms on the right-hand side of equation (1) are associated with symmetric division (proliferation rate, p_3_), cell death (death rate, d_3_), asymmetric division in previous stage 2 (differentiation coefficient, 1 − r_2_, proliferation rate, p_2_), and direct differentiation into next stage 4 (differentiation function D_3_). The terms on the right-hand side of equation (2) are determined by direct differentiation from stage 2 (differentiation function, D_3_), direct differentiation into next stage 4 (differentiation function, D_4_), and cell death (death rate, d_4_). The terms on the right-hand side of equation (3) are associated with direct differentiation from previous stage 4 (differentiation function, D_4_) and cell death (death rate, d_5_). In equations (1, 2, 3), all rates have units of time^−1^ (day^−1^) and the coefficient, 1 − r_2_, describing differentiation via asymmetric division is dimensionless. Both functions describing direct differentiation have units of number of cells/time (number of cells/day). The function f(n_tot_) (dimensionless) describes the feedback signal affecting the rates of cell number in different stages if the total cell number approaches a threshold (see also Methods section).

Each of the differentiation functions, D_3_ and D_4_, describes the actuation by the corresponding transcription factor (MyoD and myogenin, respectively) of the next myogenic factor (myogenin and MHC, respectively). It is also assumed that the two transcription factors induce the –expression of their corresponding targets by means of a signaling molecule, S generated by the application of strain (Fig. 1b). We now discuss the particular forms of the differentiation functions, D_3_ and D_4_. In equation (2), this function D_3_ is equal to the rate of the change in cell number in stage 4 due direct differentiation of a fraction of cells in stage 3. The corresponding molecular mechanism is the transcriptional activity of MyoD (expressed in stage 3) toward myogenin (expressed in stage 4) production. The change in the myogenin production occurs via binding of the transcription factor, MyoD to the myogenin promoter. The duration of this binding is much shorter than that of the resulting production of myogenin (or time of the n_4_ change in equation (2)), and D_3_ can be considered to be proportional to the probability of MyoD being bound to the promoter of myogenin which can be described by the following relashionship^24^.


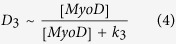


Here [MyoD] is the concentration of MyoD and k_3_ is the dissociation constant of MyoD from the promoter of myogenin (k_3_ has the units of concentration, and the right-hand side in (4) is dimensionless). If the MyoD transcription activity is pre-conditioned by the effect of the signaling molecule, S then the total [MyoD] concentration in equation (4) has to be replaced with the fraction of MyoD affected by S. Since this effect is also much faster than the change in myogenin production (and the change in n_4_) the number of MyoD molecules affected by S can be found from the steady state solution of the corresponding kinetic equation^24^. This results in the following modification of the relationship (4)


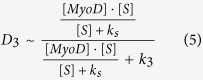


Here, k_s_ (units of concentration) is a constant characterizing S/MyoD interaction (if the mechanism of such interaction is direct binding then k_s_ is the dissociation constant), and [S] is the concentration of the signaling molecule. We now take into account that MyoD transcriptional activity takes place in all cells belonging to state 3 and re-write the right-hand site relationship (5) as


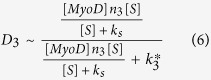


Here, 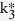 is a constant with units of number of cells × concentration that will be estimated below. Then, we assume that the concentration of signaling molecule, [S] is proportional to the applied strain ([S] = ε[S_0_] where [S_0_ ] is the concentration coefficient, and ε is the strain coefficient equal to 1 for the 10%- amplitude the applied strain). Now, we divide the numerator and denominator in the S-function in the right-hand site of equation (6) by [S_0_], and in addition, divide the numerator and denominator of the right-hand side of equation (6) by [MyoD]. This transformation results in the differentiation function, D_3_ in its final form


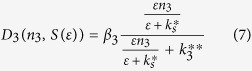


Here, β_3_ is the parameter characterizing the n_4_ production (number of cells/time, or number of cells/day), 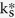 is the dimensionless parameter equal to k_s_/[S_0_], and 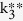 = 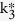/[MyoD] is a constant (units of number of cells) proportional to the dissociation constant for the MyoD binding to the myogenin promoter and inversely proportional to MyoD concentration. Both, β_3_ and 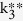, are parameters of the model whose effect and optimization will be considered below. It follows from equation (7) that for low concentrations of the signaling molecule, S (ε ≪ 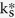), D_3_ is a linear function of n_3_, and β_3_ is the coefficient of proportionality between the two. On the other hand, for large concentrations of S (ε ≫ 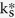), D_3_ is proportional to a sigmoidal function of n_3_, and β_3_ can be defined as the maximum differentiation level (similar to such coefficient in the equations for actuators^24^) of the cells in stage 3. Using similar arguments for the myogenin/MHC interaction and its dependence of the strain-associated signaling molecule, S, we obtain the following equation for the differentiation function D_4_.


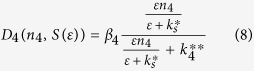


Here, β_4_ is the parameter characterizing the n_5_ production with the units of number of cells/time, or number of cells/day, and 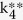 is a constant (units of number of cells) proportional to the myogenin/MHC promoter dissociation constant and inversely proportional to myogenin concentration. Similar to the β_3_ parameter, β_4_ is the coefficient of proportionality between n_4_ and D_4_ if the concentration S is low. Moreover, β_4_ has the meaning of the maximum differentiation level if the concentration, S, is high. For simplicity, we assume the same constant, 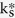, for the interaction of the signaling molecule, S with both MyoD and myogenin.

Supplementary Fig. S1 shows broad effects of the parameters, β_3_, β_4_, 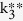, and 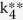, on the differentiation functions, D_3_ and D_4_. The effect of a β_3_-increase causes significant changes in cell numbers in stages 3 and 4 resulting in their sharper decrease upon reaching their respective maxima. The effects of β_3_-increase on cell number in stage 5 and the total cell number are less noticeable. An increase in β_4_, does not change the kinetics of n_3_ but affects n_4_ (by decreasing) and n_5_ (by increasing). An increase in 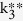, makes a significant effect on all components of the differentiation, n_3_, n_4_, n_5_, and n_tot_. In this case, the transition of cells in stage 3 into stage 4 is smaller resulting in more cells in stage 3 and fewer cells in stage 4. With the constant 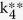 remained unchanged, the 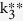-increase still affects the differentiation function, D_4_, resulting in a smaller initial slope of n_5_(t) but in larger values of n_5_ in later days. In terms of the effect of the constant, 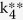, it does not affect n_3_, but larger values of 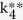 correspond to less effective differentiation into the final stage 5. Larger values of 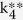 result in a smaller initial slope of n_5_(t) and smaller values of n_5_ at later times. However, the total number of cells remains approximately the same due to the balance between n_4_ and n_5_. Thus, the parameters, β_3_ and β_4_ enhance differentiation, while the constants, 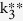, and 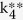, (proportional to the transcription factor/target promoter dissociation constants) inhibit it.

The concentration of signaling molecules, S, is determined by the applied strain, ε(t). Although the strain is cyclic for one hour of each day, we assume that S is the total number of signaling molecules produced during this relatively short, transient period of time that determines the signaling molecule’s interaction with transcription factors, MyoD and myogenin, for the rest of each day. The results below will be obtained assuming that S is proportional to the magnitude of the strain, ε_0_, but more general functions of strain will be discussed as well.

### Comparison with the experiment and optimization of the model parameters

There are two possible ways to compare the modeling results with the previously obtained experimental data^22^. In one approach^23^, the experimental data in terms of the myogenic factors from Huri *et al*.^22^ are converted into the corresponding values of the cell numbers in the n^th^ stage, and the results are compared with the model data in terms of the n’s. The converse approach of converting the n’s to myogenic factors is, however, more advantageous in our case because the experimental data^22^ do not include myogenin. As such, we convert the modeling data obtained in terms of n_2_, n_3_, n_4_, n_5_, and n_tot_ into the corresponding data in terms of the differentiation factors and compare them with the experimental results for Desmin, MyoD, MHC, and the total cell number.

**Figure 2:**
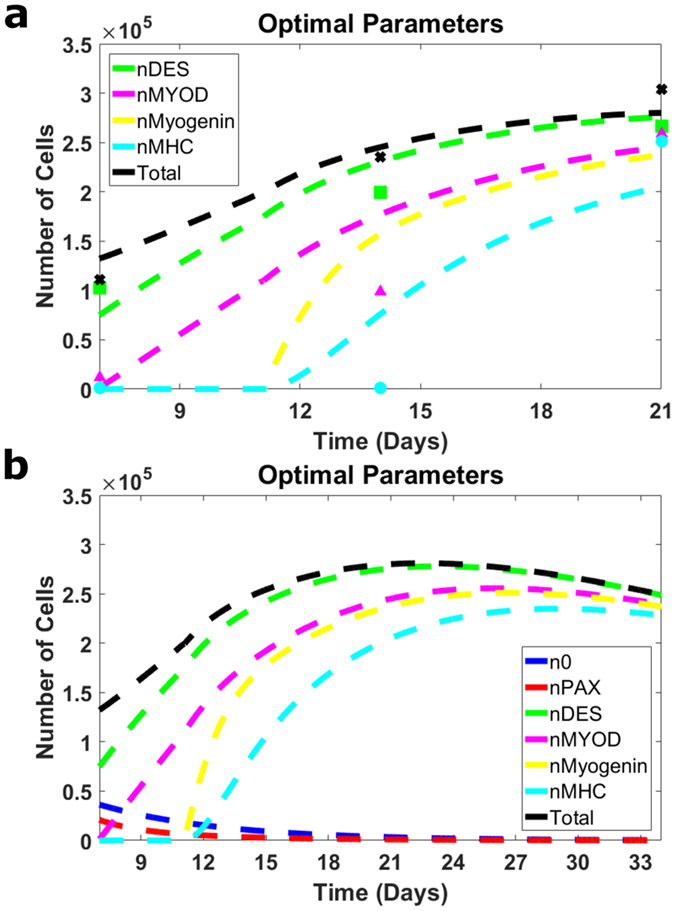
Modeling results vs. experimental data^22^ and optimization of the parameters. Here, the modeling results are presented in terms of cell numbers expressing particular factors, which is different from cell numbers, n_1_–n_5_, but has an advantage in the comparison with the experimental data. The computed total cell number is also used for the comparison with the experimental data. The modeling results are shown in dashed lines, and the experimental data for days 7, 14, and 21 and strain magnitude of 10% are shown in squares (number of cells expressing Desmin), triangles (number of cells expressing MyoD), circles (number of cells expressing MHC), and crosses (total cell number). (**a**) Computed kinetics for the optimal values of the model parameters vs. experimental data for the time interval of the differentiation part of the experiment (from day 7 through day 21). (**b**) Computed kinetics for longer period of time (through day 33).

We use the comparison with the experimental data to compute the optimal values of the model parameters, β_3_, β_4_, 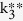, and 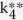 which we define as those that minimize the sum of squared differences between the experimental and modeling results at days 7, 14, and 21. The optimization is based on the comparison with experimental data for the applied strain of 10% and then used below for other values of strain. The details of the optimization method are discussed in the Methods section, and the optimal parameters are included in Table 1. In Fig. 2a, we show (in dashed lines) the time course of Desmin, MyoD, myogenin, and MHC as well as the total cell number vs. experimental data (triangles, squares, circles, and crosses for Desmin, MyoD, MHC, and total cell numbers, respectively) at days 7, 14, and 21. The kinetics of the myogenic factors is of significant interest for enhancing the understanding of the process. This can be achieved by the use of the optimized parameter and the modeling power to reveal the evolution of the differentiation factors, Desmin, MyoD, myogenin, and MHC for longer times. The chosen time interval cannot be too short (e.g., cutting the presentation at 21 days would not allow the late factor, MHC to reach its maximal point), but it should not be too long either (not to skew important details on days 7, 14, and 21). In this regard, the interval of 33 days seems reasonable, and it is also used below for the predictions of the effects of other strains. Thus, Fig. 2b presents the model-predicted kinetics of Desmin, MyoD, myogenin, MHC, and total cell number through day 33. The kinetics of the earlier factor PAX7 and that of the original stem cell number are also included for the completeness of the picture.

### Strain dependence of stem cell myogenic differentiation

**Figure 3:**
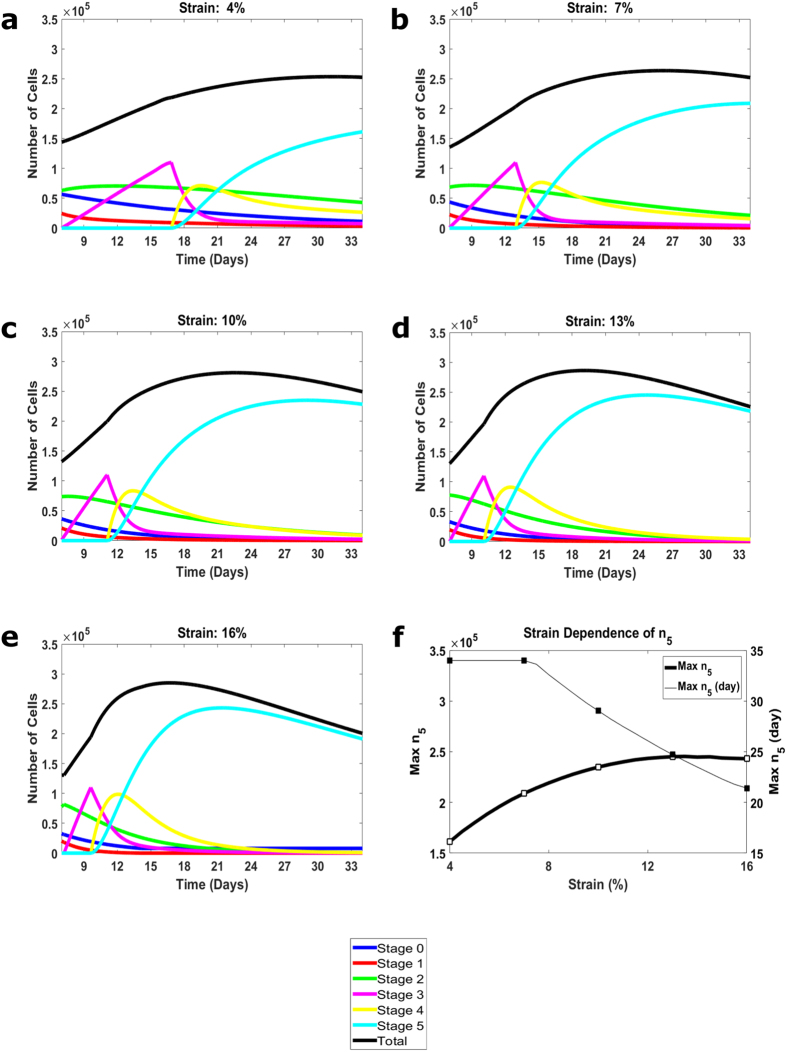
Model predictions of differentiation kinetics for different strains. (**a**,**b**,**c**,**d** and **e**) Computed functions n_1_, .., n_5_ and the total number of cells, n_tot_, for strain amplitudes of 4%, 7%, 10%, 13%, and 16%, respectively. (**f**) Maximal cell numbers in the latest stage, 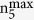 (open squares) and days of their reaching (dark squares) for strain amplitudes of 4%, 7%, 10%, 13%, and 16%.

In Fig. 3, we present the model predictions of the kinetics at different strains. Figure 3a–e show the time course of cell numbers in all 6 stages for the strain amplitudes of 4%, 7%, 10%, 13%, and 16%, respectively. Figure 3f presents the strain dependence of the maximal values of n_5_ and the times (days) of reaching these maxima. For these predictions, we use the parameters of the differentiation functions obtained from the optimization. Other parameters involved are the n_3_-and n_tot_-thresholds, and, for the consistency, they were chosen to be close to those used in the previous analysis^23^ of the earlier stages of myogenesis. The parameters used are collected in Table 1.

The strain effect on stem cell myogenesis is several-fold. We start with the analysis of cells in stage 5 expressing the latest factor, MHC which determines the outcome of myogenesis. The moment when MHC is first expressed is a function of strain, and it is reached earlier for larger strains: the earliest is day 10 for strain of 16% and the latest is day 17 for strain of 4%. Then, n_5_ exhibits an approximately linear increase with a slope which is greater for larger strains. Note, that the magnitudes of n_5_-slopes are correlated with the preceding slopes of cell number in stage n_3_ (Fig. 3a–e). At the later times, the n_5_-slopes decrease for all strains: there is still a slower increase in n_5_ for smaller strains of 4% and 7% while at larger strains of 10%, 13% and 16%, n_5_ decreases. Within the considered 33-day time interval, the number of cells expressing MHC (being in stage 5) reaches its maximal value which is greater for larger strains (1.6 × 10^5^ for the strain of 4%, 3 × 10^5^ for the strain of 16%, Fig. 3f). For smaller strains of 4% and 7%, the increases in n_5_ become slower but the function is still monotonic reaching the maximal value on day 33 (Fig. 3f). In contrast, for larger strains of 13% and 16%, the maximal value of n_5_ is reached earlier, on days 24 and 21, respectively (Fig. 3f).

All these characteristics, the initial moment of MHC expression, the initial n_5_-slope, and the maximal reached value of n_5_, change monotonically as the strain increases from 4% to 16%. Interestingly, the maximal value of n_5_ as a function of strain changes nonlinearly and appears to saturate for larger values. Such saturation is confirmed by the analysis of differentiation functions, D_3_ and D_4_ below. The observed phenomenon of saturation of the strain dependence of the solution is due to our model (equations (7) and (8)) of the differentiation functions.

We now discuss the interplay among the factors, MyoD, myogenin, and MHC involved in the differentiation process. The n_3_-function has two characteristic parts, a linearly increasing section whose slope increases with the strain and a decreasing section that behaves similarly for all strains (Fig. 3a–e). In the latter section, the decreasing function asymptotically approaches zero, and the moment when it practically disappears is reached later for smaller strains (day 27 for the strain of 4% vs. day 17 for the strain of 16%). This kinetics is important for further expression of the myogenin and MHC factors. The n_4_-kinetics looks qualitatively similar for all strains; the function first increases, reaches a maximum, and tends to zero. Quantitatively, however, the n_4_-curves for different strains differ: the maximal values are greater and the moments of disappearance are reached earlier for larger strains.

**Figure 4:**
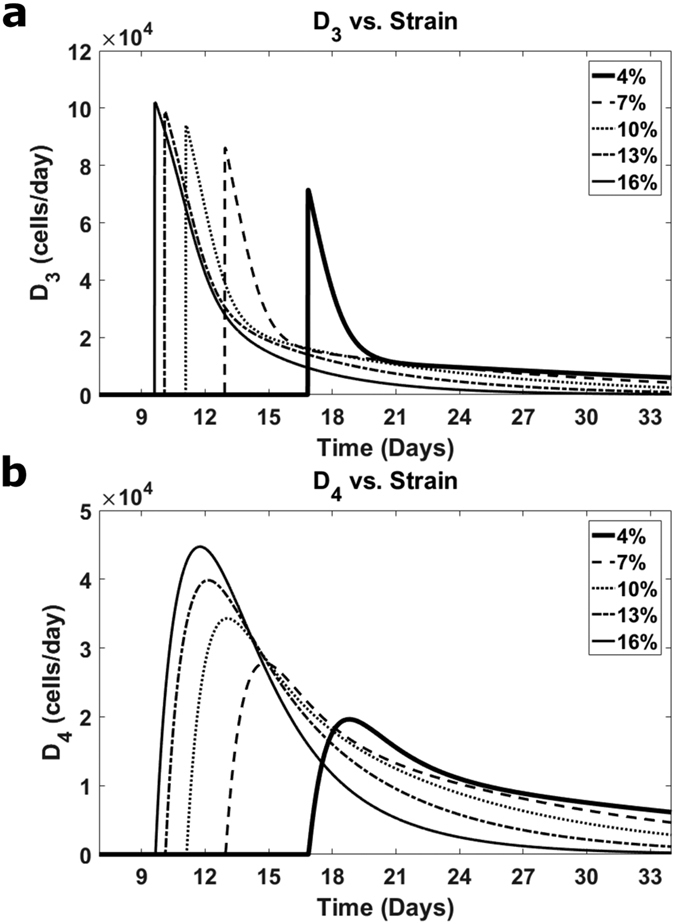
(**a** and **b**) The differentiation functions, D_3_ and D_4_, respectively, for the strain amplitudes of 4% (thick solid lines), 7% (dashed lines), 10% (dotted lines), 13% (dashed-dotted lines), and 16% (thin solid lines).

Finally, the major features of stem cell differentiation can be well-characterized by the behavior of the differentiation functions, D_3_ and D_4_ (Fig. 4). The first of these functions, D_3_, jumps from zero at a moment determined by n_3_ reaching a prescribed threshold (equal here to 1.1 × 10^5^) (Fig. 4a). Then D_3_ decreases, more gradually for larger strains, which means that the time interval of intense differentiation of stage n_3_ into stage n_4_ lasts longer for such strains. Due to the dependence of D_3_ on both S(ε_0_) and n_3_, the function, D_3_ then reaches the time where the differentiation becomes weak but it is greater for smaller strains. Our model for the strain dependence of differentiation results in the clear saturation of the D_3_-function for larger strains (see the maximal values of D_3_ for the strains of 10%, 13%, and 16% in Fig. 4a). The function, D_4_ becomes positive at the same moment as the function D_3_ does, but, in contrast to D_3_, the function, D_4_ has a time interval of a monotonic increase. Then, the differentiation function, D_4_ reaches (earlier for larger strains) its maximal value. After this, D_4_ decreases and tends to zero more sharply for larger strains (Fig. 4b). The maximal values of D_4_ monotonically increase with the strain magnitude but exhibit saturation similar to the D_3_-function. The n_3_-to-n_4_ and n_4_-to-n_5_ differentiation is strongest when D_3_ and D_4_ reach their maximal values. The maximal value of D_4_ is smaller than that of D_3_, providing a positive production rate of cells in stage n_4_ (equation (2)). In summary, the differentiation part of stem cell myogenesis starts with a rise of the cell number in stage 3 that triggers the appearance and increase in cell number in stage 4. After this, cells in stage 3 decrease as they are converted to stage 4 (n_4_ increases). The rise in cell number in stage 4 triggers the appearance and increase in cell number in stage 5. Following this, the cell number in stage 4 decreases, and finally, cells in all stages, from 0 to 4, disappear as they are differentiated into the latest stage 5. The kinetics of cells expressing particular factors is, however, different. The numbers of cells expressing Desmin, MyoD, myogenin, and MHC all increase with time (Fig. 3b) consistent with the ultimate conversion of all cells into stage 5. The discussed features of differentiation are common to all considered cases of strains above 4%, while the quantitative characteristics of the process are strain-dependent. The differentiation becomes less effective at lower strains, although we do not consider here the strains below 4%. This conclusion on the weaker myogenesis for smaller strains is consistent with the previous observations^22^,^23^ of the absence of the late myogenic factor, MHC throughout the whole time interval of the experiment if the strain is not applied.

## Discussion and Conclusions

In our new model of stem cell myogenic differentiation, we focus on the interaction among three late factors, MyoD, myogenin, and MHC. The earlier factors, PAX7 and Desmin, do not enter the differentiation functions but they affect the differentiation kinetics via the moment when n_3_ reaches the prescribed threshold and through the Desmin-related proliferation term in the n_3_-rate (equation (1)). The mechanism of direct differentiation of cells in stage 3 into stage 4 and cells in stage 4 into stage 5 is based on the transcriptional activity of the MyoD and myogenin factors. We use an approach developed in the theory of transcription networks^24^ that permits a simplification of the kinetic equations of the transcription factor/target protein interaction. This simplification takes place due the difference in time scales of the binding and transcription/translation stages of this interaction. There is a number of transcription (myogenic regulatory) factors and proteins involved the myogenic differentiation^25^. Here we reduce these two groups to MyoD/myogenin and MHC, respectively, but the model can be extended to include additional factors of differentiation. We also propose a conceptual model of the strain effect on myogenic differentiation, introducing a signaling molecule, S that affects MyoD and myogenin as a pre-condition of their transcriptional activity (Fig. 1b). It results in functions that saturate when large concentrations of S enter the differentiation functions, D_3_ and D_4_ (equations (6) and (7)). On the other hand, such functions in the model of myogenesis mean that there is no direct differentiation if the external strain is not applied. This is consistent with the experimental results^22^,^23^ for ASCs, but the model might require an extension, such as a baseline terms in functions D_3_ and D_4_, if a no-strain component of stem cell myogenesis is significant. Another question is what the identity of the hypothetical molecule S would be. Mechanotransduction signaling typically starts with integrin-mediated FAK and then involves the Rho pathway. In the case of myogenesis, these components of signaling are up-regulated in the earlier, proliferation, stages, but they become down-regulated in the later, differentiation, stages, which is necessary for exiting cell cycle and further cell fusion^26^,^27^. One more important question is how the concentration of the signaling molecule, S is related to the applied strains, including such strain features as the magnitude, frequency, and duration of the cyclic part etc. While in our model here, we limit the S-dependence on the applied strain to the strain magnitude, there are plausible scenarios where the strain frequency is involved too. Indeed, if the production of S-molecules per strain cycle is determined by the local stress or stored energy in a viscoelastic extracellular matrix then the resulting S-concentration will depend on both amplitude and frequency of the applied strain. Altogether, these motivate further experimental investigation of the molecular pathways associated with the strain effect and characteristics of the strain effect itself on stem cell myogenesis.

The model presented here differs in several important aspects from the previous version^23^: it (1) focuses on the differentiation part of stem cell myogenesis, (2) introduces myogenin, an additional transcription factor, (3) proposes biological mechanisms of the interaction of the differentiation factors, (4) also proposes a conceptual mechanism of the strain effect resulting in the theoretical prediction of the strain dependence, including strain-saturation effect, and finally (5) is based on the set of the parameters optimally fitting the experimental data.

In conclusion, we have developed a novel model of stem cell myogenic differentiation accompanied by the application of the mechanical strain. The model describes the differentiation kinetics and its association with the strain-dependent signaling. The model parameters optimally fit the experimental data for ASCs subjected to the strain of a particular magnitude, and the model results predict the stem cell behavior for different strains. The developed conceptual approach provides a better understanding of the interplay among myogenic factors involved, and it can help in the broader analysis of different stem cells and extracellular matrices (including 3-D microenvironment) used in cell therapies of skeletal muscle dysfunctions.

## Methods

### Computational Solution

Our modeling approach implements a system of ODEs and is programmed in MATLAB using the solver ode45. The initiation of late-stage differentiation in the model is marked by satisfying a stage 3 cell count threshold, 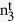. In the model, this value is set to 1.1 × 10^5^ (Table 1). Only when there exists a sufficient number of cells in this third stage will differentiation to higher stages begin.

### Feedback Factor

A feedback factor, f(n_tot_) is introduced a function of total cell number, n_tot_, total cell number threshold, 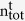, and rate constant, s, and it is given by the equation


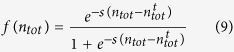


This function is multiplied to the end of each differential equation in the system of ODEs (equations (1, 2, 3)). It does not alter the kinetics of cell growth while the total cell number is less than the threshold. However, as the total cell number approaches the designated threshold, the value of the function rapidly diminishes to curb further cell growth. As a result, when the total number of cells is above the set threshold, the value of the function approaches 0. This, when multiplied into the system of ODEs leads to differentials that approach 0, thus exhibiting unreasonable steady-state behavior. To mitigate this phenomenon at higher strains, the threshold n^‡^ was set up to 4 × 10^5^.

### Optimization

In order to optimize (optimally fit the experimental data) the parameters, β_3_, β_4_, 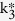, and 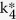, the Optimization toolbox (R2015a) in Matlab is used. The objective function targeted in the optimization is a sum of squared differences (SSD) between the modeling results and experimental data on days 7, 14, and 21. As the main tool of optimization, we use the function, Fmincon that is based on an interior point method, iterative minimization of a quadratic approximation over a smaller region defined by a line search. We double check the results of our parameter optimization using an alternative MATLAB function, lsqnonlin, which implements the Levenberg-Marquadt algorithm, a hybrid method of gradient-descent and Gauss-Newton. With the set of optimal parameters shown in Table 1, the SSD is reduced by approximately 30%.

## Additional Information

**How to cite this article**: Deshpande, R. S. and Spector, A. A. Modeling Stem Cell Myogenic Differentiation. *Sci. Rep.* **7**, 40639; doi: 10.1038/srep40639 (2017).

**Publisher's note:** Springer Nature remains neutral with regard to jurisdictional claims in published maps and institutional affiliations.

## Supplementary Material

Supplementary Information

## Figures and Tables

**Table 1:** List of parameters used.

	Parameter	Value
Thresholds	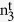	1.1 × 10^5^
	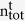	4.0 × 10^5^
Earlier Stages	p_2_	0.36
	r_2_	0.50
	d_2_	0.10
Later Stages	β_3_	1.9 × 10^5^
	β_4_	0.8 × 10^5^
	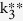	0.6 × 10^5^
	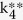	2.0 × 10^5^
	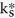	0.75
	d_3_	0.20
	d_4_ = d_5_	0.03

Optimized parameters of stem cell differentiation, β_3_, β_4_, 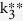, and 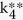, as well as other (prescribed) parameters, 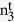 and 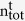 (thresholds); p_2_, r_2_, and d_2_ (earlier stages); and 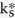, d_3_, d_4_, and d_5_.
