# Supplementary material for: Modeling Stem Cell Myogenic Differentiation
Source: Sci Rep. 2017 Jan 20;7:40639. doi: 10.1038/srep40639 (PMC5247743; doi:10.1038/srep40639)
Supplement: Supplementary Information [file srep40639-s1.pdf]

Modeling Stem Cell Myogenic Differentiation

Rajiv S. Deshpande[1] & Alexander A. Spector[1*]

[1]Department of Biomedical Engineering, Johns Hopkins University, Baltimore, MD 21205

Supplementary Information

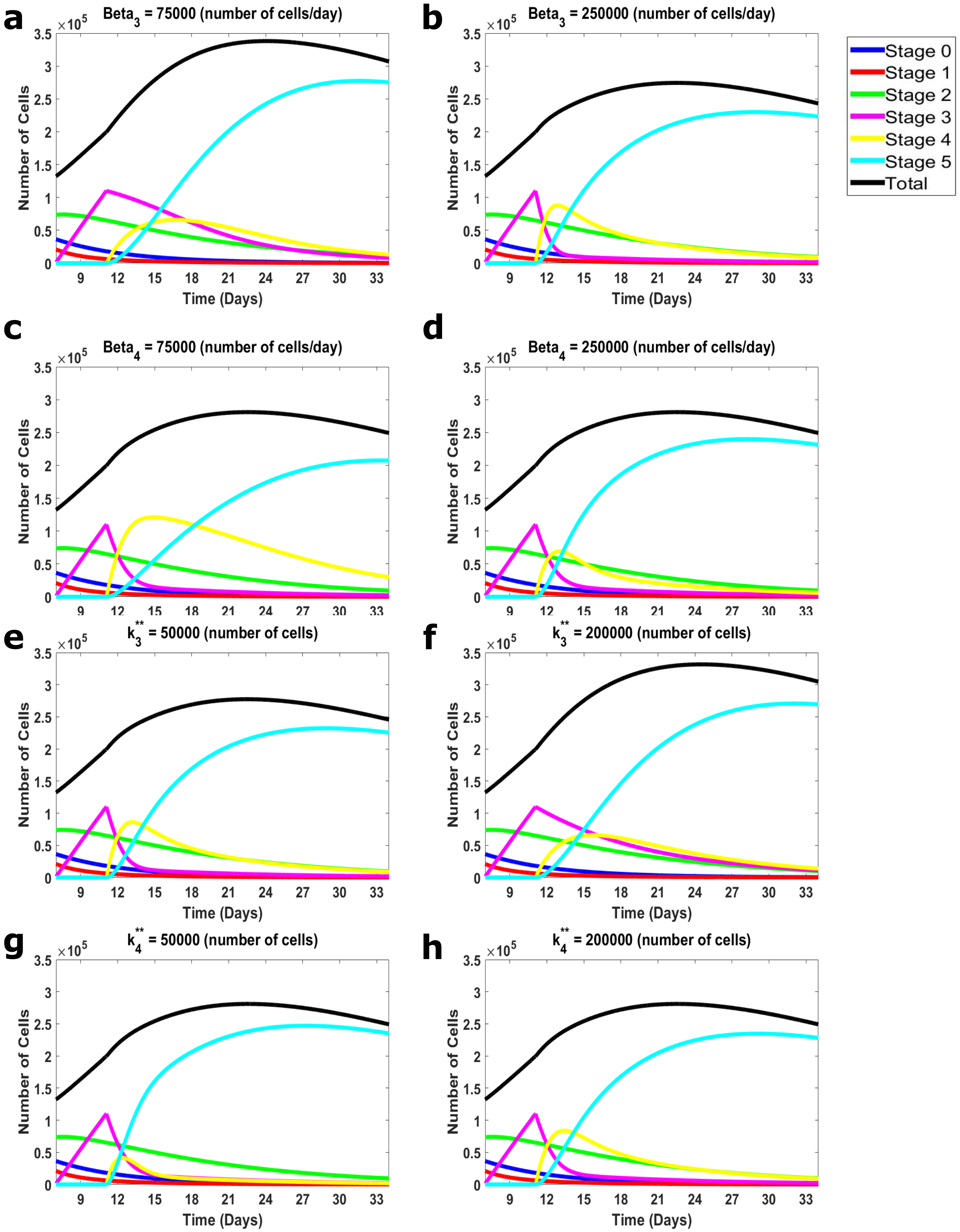
a
Beta3 = 75000 (number of cells/day)
b
Beta3 = 250000 (number of cells/day)
c
Beta4 = 75000 (number of cells/day)
d
Beta4 = 250000 (number of cells/day)
e
k3** = 50000 (number of cells)
f
k3** = 200000 (number of cells)
g
k4** = 50000 (number of cells)
h
k4** = 200000 (number of cells)
×10^5
Number of Cells
Time (Days)
Stage 0
Stage 1
Stage 2
Stage 3
Stage 4
Stage 5
Total

Figure Legend

Supplemental Figure S1. Effect of the differentiation parameters on differentiation kinetics for the strain magnitude of 10%. For each of the considered parameters, β_3, β_4, k_3^{**}, and k_4^{**}, that enter the differentiation functions, D_3 and D_4, the differentiation kinetics is computed for two, small and large values. The differentiation part of the myogenic process is considered started when the cell number in stage 3 reaches a prescribed threshold. For the completeness, the earlier stages (n_0, n_1, and n_2) are included too. The parameter, k_s^* is considered fixed. a) and b) The effect of β_3: β_3= 75,000 number of cells/day and 250,000 number of cells/day, respectively. c) and d) The effect of β_4: β_4=75,000 number of cells/day and 250,000 number of cells/day, respectively. e) and f) The effect of k_3^{**}: k_3^{**}=50,000 number of cells and 200,000 number of cells, respectively. g) and h) The effect of k_4^{**}: k_4^{**}=50,000 number of cells and 200,000 number of cells, respectively.
